# The Era “or Error” of Second Localization Procedures

**DOI:** 10.1155/tbj/6391905

**Published:** 2025-09-29

**Authors:** Nicole Nelson, Jennifer Den, Roi Weiser, Biai Digbeu, H. Colleen Silva, Angelica S. Robinson, Flavia Poselman, V. Suzanne Klimberg

**Affiliations:** ^1^Department of Surgery, University of Texas Medical Branch, Galveston, Texas, USA; ^2^Department of Surgical Oncology, University of Texas MD Anderson Cancer Center, Houston, Texas, USA; ^3^Department of Biostatistics and Data Science, University of Texas Medical Branch, Galveston, Texas, USA; ^4^Department of Radiology, University of Texas Medical Branch, Galveston, Texas, USA

**Keywords:** fluoroscopic intraoperative neoplasia detection, fluoroscopic localization, margin positivity, re-excision rate

## Abstract

**Background:** Clips placed after core needle biopsy are often several millimeters to centimeters from the biopsy cavity. Radiofrequency and radar (R) localization involve a second localization procedure based on the prior clip placement, potentially compounding the distance from the area localized to the original biopsy site. Fluoroscopic intraoperative neoplasm detection (FIND) obviates the need for a second localization by using intraoperative fluoroscopy to localize the original biopsy clip. We hypothesized that intraoperative localization using FIND is feasible and may result in fewer positive margins.

**Methods:** A retrospective review was performed of patients with nonpalpable malignancy who underwent partial mastectomy from September 2016 to August 2023. Results were compared between patients who underwent R localization vs. FIND. The Pythagorean theorem was used to calculate the distance in space between the biopsy clip and the R localization device. Chi-square was used to calculate the two-tailed *p* value.

**Results:** We identified 219 patients: 161 localized with FIND and 55 with R. Three percent (6 out of 161) of the patients with FIND and 12% (7 out of 55) of the patients with R had positive margins (*p*=0.01). The average distance between the R device and biopsy clip in patients with positive margins was 19.1 mm, and with negative margins, it was 12.45 mm (*p*=0.09).

**Conclusions:** The positive margin rate with R localization was significantly greater than with FIND. The positive margin rate trended toward increased distance from the localization device to the biopsy clip. Eliminating the second localization decreases painful procedures for the patient and may result in improved tumor-free margins.

## 1. Introduction

Since the NSABP B-06 study showed an acceptable recurrence rate for breast conservation therapy compared to mastectomy [1], surgeons have been striving to improve the process to provide the best possible outcomes and improve the patient experience. Improvements in screening mammograms since that time have allowed us to detect malignancy at smaller sizes and earlier stages, often when these lesions are nonpalpable. This has led to the development of multiple techniques to localize nonpalpable lesions intraoperatively, hoping to decrease the rates of positive margins and the need for re-excision and improve the overall patient experience. Several methods have been employed in the past. Historically, this was done with wire-guided localization (WGL), which required placement on the day of the procedure by radiology and had the potential for dislodgement [2] and the uncomfortable effect of requiring patients to have a wire protruding through their skin until surgery. Wireless localization methods have recently been popularized, including initially with radioactive seeds. However, this required the introduction of numerous policies and resources in systems that chose to adopt this method given the radioactive nature of the device placed [3, 4]. More recently, nonradioactive localization devices have been popularized, and they use various technologies to help with intraoperative localization, including radar, radiofrequency (R), and magnetic technologies [5, 6]. The benefit of these includes the ability to be placed days to months pre-operatively, which helps improve the operating room (OR) day's efficiency and removes the externally visible component of the wire to improve the patient experience. Previous studies have shown that positive margin rates for these devices are comparable to wire localization [4]. Several studies have also demonstrated an improvement in the cost-effectiveness of these methods, given the reduction in OR delays and improvements in re-excision rates [7, 8]. The downside, especially for patients and rural surgeons, is the extra procedure and extra cost of equipment needed to perform these procedures.

We questioned whether a technology widely available, inexpensive, and used by virtually every general surgeon during their training/practice, such as fluoroscopy, could be used for the same aim. Previously, we found that fluoroscopic intraoperative neoplasm or node detection (FIND) was a feasible method of intraoperative localization and had improved margin rates compared to traditional wire localization [9]. However, up to this point, no data has compared this method to non-wire-based localization technologies.

One downfall of all devices-based localization methods is the potential for device migration. It is well known that clips placed following core needle biopsy (CNB) aren't always at the exact location of the biopsy cavity but rather in close proximity. Kass et al. showed that the biopsy clip placed following CNB, on average, ends up 13.5 mm ± 1.6 mm, SEM (95% CI = 10.3 mm–16.7 mm) from the actual biopsy cavity [10]. Often, after a biopsy of small non-palpable lesions, there is no residual imaging evidence of the lesion, and localization devices are placed based on the location of the original biopsy clip. If the lesion is then localized with another device that also has the potential for placement mm to cm away from the original biopsy clip, this could effectively compound the overall distance from the area being localized to the site of the actual tumor bed. In our study, we evaluated whether FIND could be successful used to accurately locate lesions intraoperatively and potentially improve positive margin rates by eliminating a second localization procedure. We also wanted to investigate whether distance from the localization device in patients who underwent a second localization procedure correlated with a positive margin rate.

## 2. Methods

### 2.1. Patients

**Figure 1:**
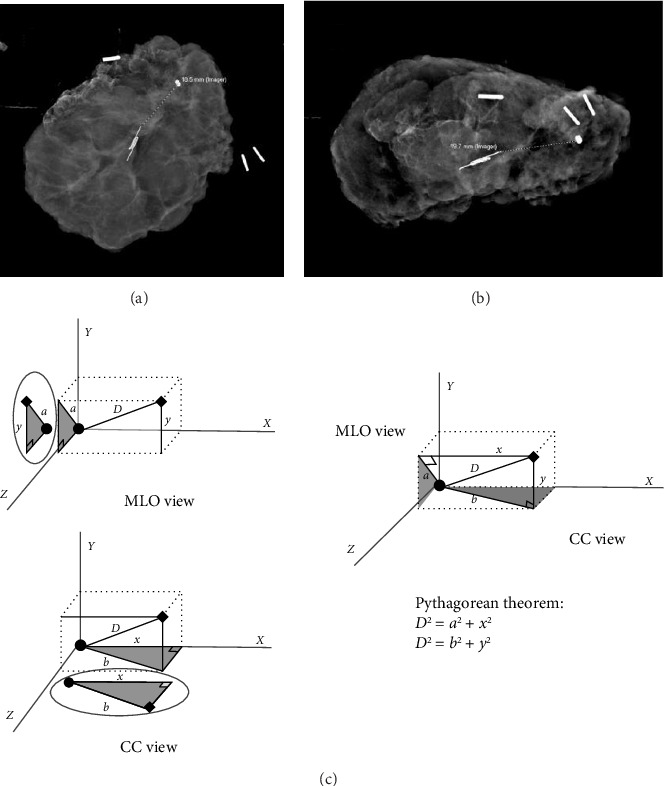
Distance from the biopsy clip to the localization device is measured in two orthogonal views. Specimens are oriented by placing sutures at the superior and lateral margins. One clip is placed on the superior margin suture and two clips on the lateral margin suture. In image (a), the superior margin is at the top of the image and lateral margin is to the right edge of the image similar to what would be seen in a medial lateral oblique view on mammogram. In image (b), the posterior margin is at the top of the image and lateral is again to the right similar to what would be seen in a craniocaudal view on mammogram. Image (c) describes how the Pythagorean theorem is used to calculate the distance in space traveled from the biopsy clip to the localization device.

This is a retrospective review of patients from a single institution. After IRB approval (#19-0185) patients were identified by querying billing software to identify a list of patients who had undergone procedures using the CPT codes 19281-19288, 19301, 19302, 19120, 19125, 19290, 19291, 76000, 76098, 76942, 77001, 77002,77021,77031,77032 between Sept 1, 2016, and August 21, 2023. A list of patients was identified. Charts were reviewed, and patients between the ages of 18 and 99 diagnosed with a non-palpable malignancy were identified. These were further reviewed to determine how their tumors were localized intraoperatively, whether by radiofrequency/radar localization or FIND. Their charts were reviewed, and information such as patient age, BMI, smoking status, presence of comorbidities, initial core biopsy, and surgical pathology, including tumor characteristics, size and margin status, and imaging, were reviewed. Imaging included an initial mammogram, ultrasound (US), and MRI if performed with localization films and intraoperative specimen mammograms. The distance between the radiofrequency and radar localization devices and the initial biopsy clip was measured in two orthogonal views on either the specimen mammogram or the localization films (if only one view was obtained of the specimen mammogram) (Figure 1).

The Pythagorean theorem was used to calculate the distance from the initial biopsy clip to the localization device based on these measurements, and this distance was recorded. We also reviewed operative results, including operative notes, operative times, fluoroscopy dosing, whether plastic surgery was involved, and indication for reoperation when performed. Data were recorded in a REDCap database.

### 2.2. FIND Procedure

**Figure 2:**
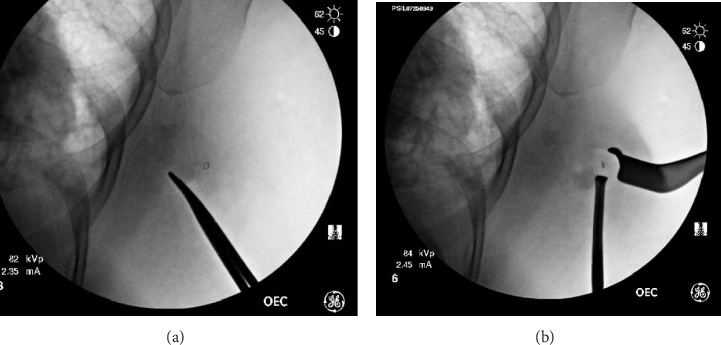
Intraoperative localization using fluoroscopy. (a) Identification of clip location and marking of the inferior border of resection using the clamp. (b) Re-evaluation immediately prior to removal of the specimen.

Our institution's radiology department uses radio-opaque clips during the initial breast biopsy. Intraoperatively, care is taken to ensure that metal bars from the OR bed or lead wires do not obscure the intended field of vision. The C-arm fluoroscope is used on quarter-dose spine settings to detect and localize the clip within the breast. Still images are used to locate the clip and mark margins and result in a minimal overall fluoroscopy time. Depth is determined by applying external pressure with a closed hemostat at varying depths from lateral to medial. The depth of the clip correlates to the depth where the clip is noted to move when external pressure is applied to the skin. Dissection can be performed with real-time feedback and a direct correlation between the C-arm images and what is visualized in the breast until the lesion containing the biopsy clip is removed (Figure 2). This can be confirmed immediately by taking a postremoval image, showing that the clip is no longer within the breast.

### 2.3. Radiofrequency/Radar Localization

Our institution used either the Hologic LOCalizerTM RFID or SAVI SCOUT Radar devices during this trial. Breast radiology placed these in standard fashion, using either US or tomosynthesis-guided localization preoperatively. Postplacement films confirmed placement, and distances from the placement device to the initial biopsy clips were noted.

### 2.4. Outcomes

Our primary outcome was the final negative margin rate. This was defined as no tumor on ink for malignant lesions and > 2 mm margins for DCIS as defined by the margin consensus statements from the Society of Surgical Oncology–American Society for Radiation Oncology–American Society of Clinical Oncology Consensus Guideline published in 2014 and 2016, respectively [11, 12]. Secondary outcomes included reoperation rate, postoperative complications, and OR time.

### 2.5. Statistical Analysis

Chi-square was used to calculate the two-tailed *p* value between the groups with respect to positive margin rate, neoadjuvant chemotherapy rates between the groups, whether shave margins were performed, and whether plastic surgery was involved. Fischer's exact test was used to determine the difference between the FIND and R groups with respect to lesion pathology, lesion laterality, and number of lesions in the specimen. Student's *T*-test was used to evaluate the age differences, number of positive nodes, tumor size, OR time, and distance from the R device to the biopsy clip.

## 3. Results

Patients underwent partial mastectomy for nonpalpable malignancy from the dates of September 1, 2016 to August 2023. We identified 161 patients who underwent localization using FIND during that time and 55 patients who underwent radiofrequency or radar localizations (R). Six patients with malignant tumors localized using FIND had positive margins (3.7%). In contrast, seven patients with tumors localized using radiofrequency or radar had positive margins (12.7%) (*p* value = 0.01). We also identified 8 out of 119 patients (6.7%) in the FIND group who underwent reoperation, six for positive margins, and two for SLN biopsy after they were found to have invasive ductal carcinoma on surgical pathology when the initial CNB had identified only DCIS. In the R group, 7 out of 49 patients (14.3%) underwent reoperation. Six underwent reoperation for positive margins, and one underwent SLN biopsy after she was found to have an invasive disease on the final pathology when CNB had only shown DCIS. One patient with < 2 mm margin for DCIS elected not to undergo re-excision.

**Figure 3:**
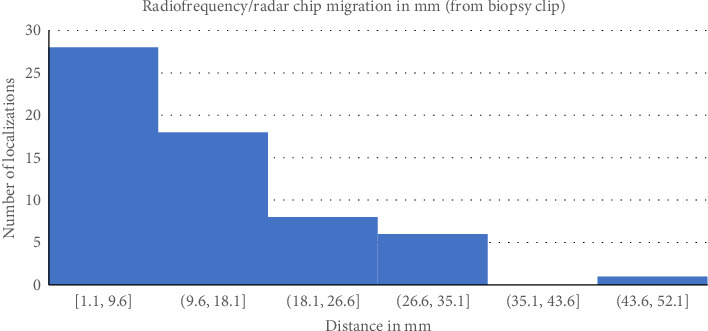
Variation in localization chip migration.

All patients during this timeframe and included in this analysis had their original biopsy clip removed, and this was confirmed on specimen mammogram. We compared the distances from the R device to the biopsy clip in patients with positive and negative margins. The average distance from the localization device to the biopsy clip for patients with positive margins was 19.2 mm (SD 10.3). For patients with negative margins, the average distance from the localization device to the biopsy clip was 12.5 mm (SD 12.5). One patient was noted to have clip migration at the time of localization, and these accounted for the distance of 44.4 mm in the negative margin group and were not used to localize the lesion (Figure 3).

### 3.1. Patient and Lesion Characteristics

There was no statistically significant difference in patient age or tumor characteristics between those who were localized with FIND versus R. The mean age was 63.4 (SD 12.1) in the FIND group and 59.9 (SD 15.6) in the R group. There was no statistically significant difference in the type of tumor localized in the FIND versus R groups, with 23 (14.3%) and 14 (25.5%) being pure DCIS, which was not statistically significant (*p*=0.06); 89 (55.3%) and 33 (60.0%) being IDC with or without DCIS; and 7 (4.4%) and 6 (10.9%) being invasive lobular carcinoma. Tumor size was similar between the groups, with the average size in mm in the FIND group at 11.76 mm (SD 9.58) and 13.8 (SD 13.6) in the R group (*p*=0.33). Clinical node positivity was similar between groups, with a number of positive nodes averaging 0.4 (SD 1.2) in the FIND group versus 0.5 (SD 1.6) in the R group (*p*=0.52) (Table 1).

### 3.2. Treatment Characteristics

There was a significant difference in the number of patients who had undergone neoadjuvant chemotherapy, with 11 (22.5%) having received NAC in the R group versus 16 (10.1%) in the FIND group (*p* value 0.02). There was no difference in the number of lesions within the specimen between the groups. Only one lesion was identified within the specimen in 96.27% of the FIND group and 90.91% of the R group. Three patients in the FIND group (1.86%) and one patient in the R group (1.82%) had three lesions identified within the specimen. Two fellowship-trained breast surgeons performed the surgery. The localization method was performed per the surgeon's preference, with one surgeon performing almost exclusively FIND and the other performing a combination of FIND and R localizations. Of the surgeons who performed the FIND procedure, there was no intersurgeon difference in the rate of positive margins for the FIND procedure. There was no difference in the use of shave margins between the groups, with 85.1% (137 of 161) of the patients in the FIND group and 85.5% (47 of 55) of the patients in the R group having shave margins performed. There was a statistically significant difference in the OR time in minutes, with the R group having a mean OR time of 128.4 (SD 64.33) minutes and the FIND group having a mean OR time of 150.1 (SD 85.77) minutes (*p*=0.05) (Table 2).

### 3.3. Primary Outcome

Final pathology identified the final positive margins in six of the FIND group, with a positive margin rate of 3.7%. In the R group, seven patients had final positive margins or a 12.7% positive margin rate, which was statistically significant (*p*=0.02). Due to the small number of patients with final positive margins, multivariate analysis could not be performed.

### 3.4. Secondary Outcomes

Reoperation rates were found to be eight in the FIND group (4.9%) versus seven in the R group (12.7%) (*p* value = 0.05). In the FIND group 2, patients had invasive cancers on their initial surgical pathology after CNB showed DCIS, and they returned to the OR for sentinel lymph node biopsy. In the R group, one patient who had DCIS with a single margin of less than 2 mm elected not to return to the OR after multidisciplinary discussions. Another patient who initially had DCIS on CNB but invasive cancer on surgical pathology returned to the OR for SLN biopsy. For the patients in the R group, we wanted to evaluate whether the distance between the R localization device and the initial biopsy clip changed the positive margin rate. Overall, the range for device migration was between 1.1 mm and 44.4 mm. The patient with a 44.4 mm migration was one in whom the initial biopsy clip was known to be displaced from the initial lesion. This patient had negative final margins. For patients with positive margins localized by R, the average distance from the biopsy clip to the localization device was 19.2 mm, with a standard deviation of 10.3. For patients localized with R with final negative margins, the distance between the initial biopsy clip and the R localization device was 12.5 mm, with an SD of 9.6. While there was a trend toward a larger distance from the biopsy clip to the R device in patients with positive margins, this was not statistically significant. The large SD was secondary to the patient with a known biopsy clip displacement with a considerable distance between the biopsy clip and device but with negative margins. In this case, the intraoperative localization was done based on the R device location, not the clip location.

## 4. Discussion

The positive margin rate in the R group was similar to historical rates for needle localization at 12%, which is significantly greater than the positive margin rate in the FIND group at only 3%. The lower positive margin rate also led to a lower reoperation rate using the FIND procedure at 6% versus 14% for R device localization, which was statistically significant. Patients in the R group with positive margins tended to have a larger distance between the R localization device and the initial biopsy clip than those with negative margins. This highlights the compounding issue that a second device localization may have the potential to increase the distance from the instrument used for localization and the true tumor bed, which plays a role in the rate of positive margins. The only tumor or operative differences that were statistically significant between the groups were the trend for more neoadjuvant therapy use in the R group and longer OR times by an average of 22 min in the FIND group. There were no statistically significant differences between the two groups in other factors traditionally attributed to the possibility of a higher positive margin rate, such as pure DCIS, invasive lobular carcinoma, or use of shave margins.

As screening techniques have improved, the majority of newly diagnosed breast cancers are nonpalpable. Various techniques have been employed to localize these tumors. Wire localization was the gold standard in past years, but this is now being replaced by other methods performed before surgery, improving OR time utilization and coordination between surgery and radiology. However, this still requires patients to undergo an additional procedure before surgery, one in which they are awake and still have the associated discomfort and cost involved in doing this. Fluoroscopy is available in virtually every system, and every general surgeon with or without additional training in breast utilizes fluoroscopy during training and in practice; thus, this technique should be easily adaptable to any system, especially rural or low-resource settings. Hayes et al. found that R localization techniques reduced OR start delays from averaging 40 min with WGL to 11 min with R localization, which results in the overall cost savings to the system [7]. FIND should have no delays as it does not require a preoperative localization procedure and thus improves OR efficiency, overall cost, need for re-excision, and patient experience. Another advantage of using the FIND procedure over radar or radiofrequency localization is that there is no limitation on tumor depth, as several studies have shown with R devices, which are difficult to localize at depths greater than 4.5 cm. Previous studies have also shown issues with potential device damage due to electrocautery, causing certain radar devices to short out and stop working, which can lead to difficulties in localization [13, 14]. There is no such issue with FIND as the target being localized does not send out a signal and cannot be deactivated by cautery.

### 4.1. Limitations

The limitation of this study is that it was a single-institution retrospective study. While there was no intersurgeon difference in positive margin rates for the FIND procedure, it only evaluated the results from two clinically active surgeons with a practice dedicated 100% to breast surgery. Results may vary for surgeons who do not dedicate this practice volume to breast surgery. As with every new technique, a learning curve is associated with using the FIND procedure. Previous studies have indicated that the learning curve for nonwire-localized technologies is around 5–7 cases [15]. We do not believe that the learning curve for FIND localization is any different, although the exact number of cases to complete the learning curve has yet to be studied. Another limitation is that institutional practices may differ in their use of clips as some are more radiolucent (bar, ring or coil shape) than others (ribbon).

### 4.2. Implications

Ideally, once more surgeons become comfortable using this technique, we would like to evaluate the performance of the FIND technique compared to R localization techniques in a prospective randomized multi-institutional study. For now, we believe this technique could quickly and easily be adopted into the repertoire of a general or a dedicated breast surgeon, even in a low-resource rural setting. This technique allows for improvement in the positive margin rate and the need for re-excision. It also has the potential to decrease the cost of the system, improve efficiency for the surgeon, and improve the overall experience for the patient.

## 5. Conclusion

FIND is a novel method of intraoperative localization that obviates the need for preoperative procedures to localize tumors. Intraoperative fluoroscopy to localize the initial biopsy clip decreases the potential for compounding errors due to increased distances from the localization device to the tumor bed. This also makes for a very time and cost-efficient method to localize tumors as there should be no intraoperative delays while awaiting device placement, and it requires no purchasing of additional localization systems or devices and relies only on the standard C-arm fluoroscopy that is already available in virtually every OR system.

## Figures and Tables

**Table 1:** Patient characteristics.

	FIND (*N* = 161) *N* (%)	R (*N* = 55) *N* (%)	*p* value
Age	63.45	59.96	0.13
Pure DCIS	23 (14.29)	14 (25.45)	0.06
Invasive lobular	7 (4.35)	2 (3.64)	1
Neoadjuvant chemotherapy	16 (10.13)	11 (22.45)	0.02^∗^
Number of positive, nodes (SD)	0.37 (1.25)	0.54 (1.62)	0.52
Tumor size (mm), mean (SD)	11.76 (9.58)	13.79 (13.60)	0.33

*Note:* R, radar and radiofrequency.

Abbreviations: DCIS, ductal carcinoma in situ; FIND, fluoroscopic intraoperative neoplasia detection.

^∗^More patients in the R group underwent neoadjuvant chemotherapy.

**Table 2:** Operative characteristics.

	FIND (*N* = 161) *N* (%)	R (*N* = 55) *N* (%)	*p* value
Shave margins performed	137 (85.09)	47 (85.45)	0.98
Plastic surgery involved	16 (9.94)	13 (23.64)	0.01^∗^
OR time (min), mean (SD)	150 (85.77)	128.40 (64.33)	0.05^∗^

*Note:* R, radar and radiofrequency.

Abbreviations: FIND, fluoroscopic intraoperative neoplasia detection; OR, operating room.

^∗^More patients in the R group had plastic surgery involvement. Operative time was longer in the FIND group by an average of 22 minutes.

## Data Availability

Data are available on request from the authors.

## Nomenclature

FIND: Fluoroscopic intraoperative neoplasia detection
R: Radiofrequency/radar
